# AKR1C3 Inhibition Therapy in Castration-Resistant Prostate Cancer and Breast Cancer: Lessons from Responses to SN33638

**DOI:** 10.3389/fonc.2014.00162

**Published:** 2014-06-23

**Authors:** Wen Zhou, Patrizia Limonta

**Affiliations:** ^1^Braman Family Breast Cancer Institute, University of Miami Sylvester Comprehensive Cancer Center, University of Miami Miller School of Medicine, Miami, FL, USA; ^2^Department of Pharmacological and Biomolecular Sciences, Università degli Studi di Milano, Milan, Italy

**Keywords:** AKR1C3, SN33638, castration-resistant prostate cancer, breast cancer, testosterone, 17β-estradiol

In the current issue of *Frontiers in Oncology* – *Cancer Endocrinology Section*, Yin et al. report that the aldo-keto reductase family 1 member C3 (AKR1C3) inhibitor SN33638 was able to reduce testosterone production, PSA expression, and cell proliferation only in some castration-resistant prostate cancer (CRPC) cells, overexpressing the enzyme, but not the majority of a panel of prostate and breast cell lines investigated, and revealing a novel caveat in developing AKR1C3 inhibitors for cancer therapy.

Prostate and breast cancers are the most common cancer and the second-leading cause of cancer-related deaths in males and females, respectively (1, 2). The mainstay treatment for advanced or metastatic prostate or ER-positive breast carcinoma is androgen-deprivation therapy or anti-estrogen therapy. Although initially these treatments are beneficial and reduce tumor burden, tumors ultimately become resistant to these therapies. There are few treatment options for this stage of cancers (termed CRPC and tamoxifen-resistant breast cancer). Thus, new approaches to treat or prevent these hormone-insensitive cancers or their progression are in great need.

The AKR1C3, also known as 17β-hydroxysteroid dehydrogenase type 5, is capable to produce intratumorally testosterone and 17β-estradiol by reducing the androgen precursors and estrone, respectively. The local conversion of less potent hormones to more potent ones will lead to nuclear receptor activation and tumor progression. Therefore, AKR1C3 has recently been regarded as a potential anti-cancer drug target in both CRPC and ER-positive breast cancer (3, 4).

The AKR1C3 inhibitor SN33638 is a lead compound newly synthesized by Dr. Jamieson’s group (5). It had low nanomolar potency against AKR1C3 and >300-fold selectivity for AKR1C3 over the other AKR1C isoforms. Equipped by this powerful compound, Yin et al. (6) treated a panel of CRPC and ER-positive breast cancer cell lines, in the presence of hormone or prostaglandin precursors, prior to evaluation of cell proliferation and levels of 11β-prostaglandin F2α (11β-PGF2α), testosterone production, PSA expression, and cell proliferation (6). They observed that *AKR1C3* mRNA was upregulated in CRPC in a meta-analysis of patient samples. 11β-PGF2α and testosterone levels in the cell line panel correlated with AKR1C3 protein expression. SN33638 prevented 11β-PGF2α formation in cell lines that expressed AKR1C3, but only partially inhibited testosterone formation and subsequent cell proliferation and/or PSA expression only in high (LAPC4 AKR1C3) or moderate (22RV1) AKR1C3-expressing cell lines. This may be explained by that there is AKR1C3-independent steroid hormone production in most cells, and the data suggest that AKR1C3 is not an effective drug target in most CRPC cancer patients.

For ER-positive breast cancers, their finding on AKR1C3 inhibition is even less promising. They observed *AKR1C3* mRNA was downregulated in patient samples, a poor correlation between AKR1C3 protein expression and 17β-estradiol production, while SN33638 was largely ineffective at preventing estrone reduction or cell proliferation. These results call into question of the possibility of using AKR1C3 inhibitors in treating breast cancer patients.

In summary, Yin et al. (6) revealed a novel finding that AKR1C3 inhibitor therapy is unlikely to be beneficial for the treatment of most CRPC and ER-positive breast cancers. Nevertheless, given the role of AKR1C3 in intratumoral steroidogenesis, we still hold enthusiastically the hope that a subpopulation of CRPC patients with high AKR1C3 might benefit from AKR1C3 inhibitor therapy, whose tumors are “addicted” from AKR1C3 to produce testosterone. The study by Yin et al. (6) provides a valuable framework for future preclinical or clinical studies aiming at verifying this hypothesis that AKR1C3 inhibition suppresses tumor formation in a selected population of AKR1C3-high CRPC patients.

## Conflict of Interest Statement

The authors declare that the research was conducted in the absence of any commercial or financial relationships that could be construed as a potential conflict of interest.
